# Large-Scale Validation of the Paddling Pool Task in the Clockmaze for Studying Hippocampus-Based Spatial Cognition in Mice

**DOI:** 10.3389/fnbeh.2019.00121

**Published:** 2019-06-07

**Authors:** Roman Sankowski, Tomás S. Huerta, Rishi Kalra, Toby J. Klein, Joshua J. Strohl, Yousef Al-Abed, Sergio Robbiati, Patricio T. Huerta

**Affiliations:** ^1^Laboratory of Immune & Neural Networks, Institute of Molecular Medicine, Feinstein Institute for Medical Research, Northwell Health, Manhasset, NY, United States; ^2^Institute of Bioelectronic Medicine, Feinstein Institute for Medical Research, Northwell Health, Manhasset, NY, United States; ^3^Elmezzi Graduate School of Molecular Medicine, Feinstein Institute for Medical Research, Northwell Health, Manhasset, NY, United States; ^4^Faculty of Medicine, Institute of Neuropathology, University of Freiburg, Freiburg, Germany; ^5^Department of Molecular Medicine, Zucker School of Medicine at Hofstra/Northwell, Manhasset, NY, United States

**Keywords:** spatial cognition, spatial memory, spatial strategy, hippocampus, Morris water maze, cognitive impairment, muscimol, Ethovision

## Abstract

Rationally designed behavioral tests are important tools to assess the function of specific brain regions. The hippocampus is a crucial neural substrate for spatial cognition, and many studies have linked hippocampal dysfunction with defects on spatial learning and memory in neurological conditions ranging from Alzheimer’s disease to autoimmune syndromes, such as neuropsychiatric lupus. While our understanding of hippocampal function, from the molecular to the system levels, has increased dramatically over the last decades, this effort has not yet translated into efficacious therapies for cognitive impairment. We think that the availability of highly validated behavioral paradigms to measure cognition in mouse models is likely to enhance the potential success of preclinical therapeutic modalities. Here, we present an extensive study of the paddling pool task (PPT), first reported by Deacon and Rawlins, in which mice learn to escape from shallow water through a peripheral exit in a circular arena dubbed the clockmaze. We show that the PPT provides highly reliable results when assaying spatial cognition in C57/BL6 mice (120 males, 40 females) and BALB/c mice (40 males, 90 females). Additionally, we develop a robust algorithm for the assessment of escape strategies with clearly quantifiable readouts, enabling fine-granular phenotyping. Notably, the use of spatial strategy increases linearly across trials in the PPT. In a separate cohort of mice, we apply muscimol injections to silence the dorsal CA1 region of the hippocampus and show that the use of the spatial strategy in the PPT relies on the integrity of the dorsal hippocampus. Additionally, we compare directly the PPT and the Morris water maze (MWM) task in C57/BL6 mice (20 males, 20 females) and BALB/c mice (20 males, 20 females) and we find that the PPT induces significantly lower anxiety, exhaustion and hypothermia than the MWM. We conclude that the PPT provides a robust assessment of spatial cognition in mice, which can be applied in conjunction with other tests, to facilitate hypothesis testing and drug development to combat cognitive impairment.

## Introduction

The hippocampus is a brain region that integrates large numbers of cortical and subcortical inputs and contributes to the neural substrate of episodic learning and memory, spatial navigation, contextual recall and emotional memory (Eichenbaum, 2000; Morris, 2007; O’Keefe, 2007; Fanselow and Dong, 2010; Hartley et al., 2014; Calhoon and Tye, 2015; Moscovitch et al., 2016). There is an emergent understanding of the different processes that give rise to these cognitive faculties at the network, cellular and synaptic levels. Moreover, it is clear that hippocampal dysfunction is a core syndrome in an array of neurodegenerative and immunological disorders, such as Alzheimer’s, chronic depression, schizophrenia, epilepsy, hypertension, Cushing’s disease, head injury, post-traumatic stress disorder, brain inflammation, cerebral hypoxia, neuropsychiatric lupus, and severe sepsis (Terry et al., 1991; Starkman et al., 1992; Aleman et al., 1999; Capuron et al., 1999; Austin et al., 2001; O’Brien et al., 2003; Elger et al., 2004; Appenzeller et al., 2006; Reitz et al., 2007; Iwashyna et al., 2010; Omalu et al., 2010; Yaffe et al., 2010; Davydow et al., 2013). All of these conditions are known to produce cognitive impairment of varying degree and long-term disability in patients. Therefore, it is not surprising that an enormous effort has been devoted to examining impaired cognition in animal models that mimic key aspects of these diseases (Xiong et al., 2013; Stepanichev et al., 2014; Huerta et al., 2015; Nazem et al., 2015; Jankowsky and Zheng, 2017; Flandreau and Toth, 2018). In particular, genetic engineering has facilitated the creation of transgenic mouse models of human diseases for which cognitive impairment is an expected behavioral phenotype (Lipp and Wolfer, 1998; D’Hooge and De Deyn, 2001; Janus, 2004; Chang et al., 2006; Lindgren and Dunnett, 2012; Jankowsky and Zheng, 2017; Honeycutt and Garcia, 2018).

Despite these advances, the drug development efforts aimed at stopping or decelerating the damage in cognition have met with little success (Hånell and Marklund, 2014), which can be partly explained by insufficient understanding of the underlying neural mechanisms. An important consideration is a need for highly dependable neurocognitive tasks in mouse models. In this regard, the Morris water maze (MWM) has been extensively used for evaluating spatial cognition in rodents. Notably, the seminal work by Morris et al. (1982, 1986) and Morris (1984) demonstrated, in rats, that place navigation in the MWM requires intact hippocampal function. The MWM is simple to implement in rats as it needs no pre-training, it is acquired relatively quickly, it incorporates a probe test that measures retention (spatial bias) and it excludes unwanted olfactory cues. Video tracking allows a fine-grain analysis of the swim paths to delineate spatial strategies, as well as potential motoric or motivational deficits. When researchers began assessing mice in the MWM, they noted that their performance was enormously variable when compared to rats. Therefore, the assay has been modified to mitigate mouse-specific problems, such as high anxiety, physical exhaustion, hypothermia, floating, and other factors that could interfere with performance in the MWM (Upchurch and Wehner, 1988; Francis et al., 1995; Wolfer et al., 1998; Hölscher, 1999; Pompl et al., 1999; Chapillon and Debouzie, 2000; Frick et al., 2000; Yoshida et al., 2001; Stavnezer et al., 2002; Wright et al., 2004; Salomons et al., 2012; Weitzner et al., 2015). When the task is optimized for mice, it is clear that certain strains perform better than others. For instance, C57/BL6 (C57 hereafter) animals are identified as good learners (Upchurch and Wehner, 1988; Wright et al., 2004; Wahlsten et al., 2005; Patil et al., 2009), whereas BALB/c mice appear to be very poor learners in the MWM (Upchurch and Wehner, 1988; Chapillon and Debouzie, 2000). The Barnes maze is another test for spatial cognition that was originally used for testing rats (Barnes, 1979) and it has also proven problematic to implement in mice, with low motivation being a potent interfering factor (Harrison et al., 2006; O’Leary and Brown, 2009; O’Leary et al., 2011).

This study validates the paddling pool task (PPT) as a mouse-specific cognitive paradigm. As stated by Deacon and Rawlins (2002), mice have a natural tendency to run into dark tunnels, especially when escaping a stressful environment such us shallow cold water. Following this notion, Deacon and Rawlins designed the circular version of the PPT, by cleverly combining aspects of the MWM and the Barnes maze (Deacon and Rawlins, 2002; Schmitt et al., 2004; Deacon, 2013). An analogous setup has shown good sensitivity for aging-associated cognitive decline (Koopmans et al., 2003). We have tested BALB/c and C57 mice and found that female and male animals of both strains learn to solve the PPT using spatial strategies and without performance differences. Notably, pharmacological disruption of the dorsal hippocampus disturbs the acquisition of spatial strategies. We establish the PPT as a robust tool for assessing hippocampus-based learning in mice, which will likely facilitate preclinical drug development for diseases associated with hippocampal dysfunction.

## Materials and Methods

### Ethical Statement

All animal experimentation was performed in accordance with the National Institutes of Health (NIH) Guidelines, under protocols approved by the Institutional Animal Care and Use Committee (IACUC) of the Feinstein Institute for Medical Research. Our Animal Research Program is registered with the Department of Health and Human Services (DHHS), Office of Laboratory Animal Welfare (OLAW), United States Department of Agriculture (USDA #21R0107), Public Health Service (PHS #A3168-01) and New York State Department of Health (NYSDOH #A-060).

### Animals

C57 (160 males, 70 females) and BALB/c (70 males, 120 females) mouse strains were used for this study (3–4 months, Jackson Laboratories, Bar Harbor, ME, USA). Covariates such as sex, litter size (*n* = 5 mice per cage), cohort size (*n* = 10 mice tested at a time) and cage enrichment were kept constant across all groups tested. Thus, each cohort corresponded to animals of the same sex and strain. Animals lived on a reverse light-dark cycle (lights off at 09:00 h, lights on at 21:00 h), for at least 14 days before testing started, with *ad libitum* access to chow and water. Experiments were performed during the dark phase of these nocturnal animals. Previous studies have shown that behavioral testing in rodents during the dark phase of the circadian cycle, when they are naturally active, leads to consistent performance (Valentinuzzi et al., 2004; Beeler et al., 2006; Roedel et al., 2006; Smarr et al., 2014). Our general approach to behavioral assessment has been described previously (Chang et al., 2006, 2009; Vingtdeux et al., 2016). All experiments occurred between 10:00 h and 18:00 h. Prior to formal testing, animals were handled for a total of 45–50 min (15 min per day on three consecutive days, or 10 min per day on 5 days), a practice we have found to be essential to lower the animals’ innate anxiety toward the experimenters.

### Transport of Animals and Configuration of the Experimental Space

Mice were brought from the colony to the laboratory in their home cages, 20–30 min before testing started, through a corridor that was illuminated with red lighting. The cages were placed in a cart inside a holding area, which was separated from the testing area with an opaque curtain. The holding area (6 m × 4 m) was dimly illuminated, in the range of 70–90 lux, with incandescent lights and contained a computer running the video software, and a “drying” station consisting of a standing heat lamp (model IR300, KS Choi, Los Angeles, CA, USA) positioned on top of a plastic cage where the mice were placed after running each trial. The testing area (4 m × 4 m) contained the clockmaze sitting at the center of a round table, 1 m from the floor (Supplementary Figure S1A). An infrared-sensitive video camera was mounted above the maze, which was connected to the video tracking system (see below). Three large objects, consisting of three-dimensional facemasks, served as prominent distal cues and were placed at ~1 m from the edge of the maze. The testing area was illuminated at ~40 lux level by floor lights that were pointed to the distal cues from below.

### Equipment Setup

**Figure 1:**
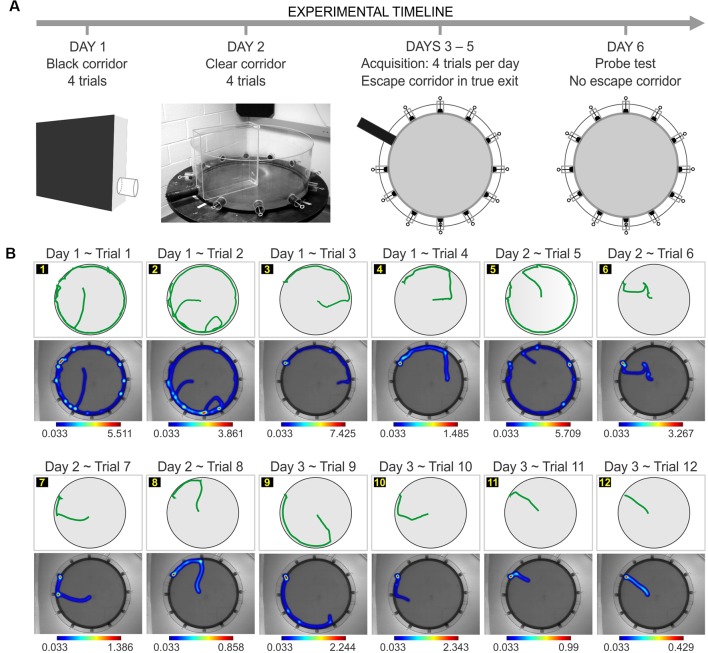
Implementation of the paddling pool task (PPT) in the clockmaze. **(A)** Experimental timeline for the task with pre-training in a black corridor (day 1) and a clear corridor (day 2). This is followed by acquisition in the clockmaze, with a single open hole (true exit; days 3–5), and a probe test with the all the exits blocked (day 6). **(B)** Representative experiment showing the trial-by-trial performance of an individual C57 female in which the true exit is at hole 10. For each trial, the track plot is at the top, and the heatmap of occupancy is at the bottom with its corresponding color scale (values are expressed in seconds).

The apparatus for behavioral testing was the clockmaze (Figure 1A, Supplementary Figures S1A,B), which consisted of a circular base platform (diameter, 85 cm), surrounded by a clear wall (30 cm high), and sealed to the base by aquarium sealant to make it waterproof. Cold water (18° ± 1°C) was added to a depth of 2 cm, sufficient to wet the underside of the belly of mice, but shallow enough to allow the animals to constantly touch the floor of the pool and paddle through the water. The perimeter wall was pierced by 12 holes, 4 cm in diameter, arranged equidistantly around the circumference so that, from the top, they appeared as the 12 h on a clock face. The lower edge of each hole was 2.5 cm above the maze floor, that is, at mouse head level. Eleven of these tubes were sealed with black plugs, flush with the internal pool wall surface; one was open and led to a dry escape pipe, which was 4 cm in diameter, made of black flexible plastic. Importantly, from within the clockmaze, the true exit looked similar to the decoys, even to the human eye.

### Video Tracking

It was conducted with dedicated software (Ethovision XT11, Noldus Information Technology, Leesburg, VA, USA; Noldus et al., 2001), which automatically detected the contours of the animal from a live video feed, distinguished it from the background, and tracked activity with a 3-point detection algorithm (video rate, 30 frames per second). Moreover, Ethovision allowed the experimenter to organize the whole experimental procedure by using trial control modules (Supplementary Figures S1B,C).

### Pre-training

Prior to testing, animals underwent pre-training on two consecutive days. The purpose was to familiarize the mice to the procedural aspects of the task, such as navigating in the cold water (instead of simply staying immobile), entering into the open hole and the adjacent pipe as a means of escape, and staying inside the pipe while being transported to the drying station. On day 1, mice experienced the black corridor (Figure 1A), which had the base and three sides made of black painted wood (30 cm long, 8 cm wide, 26 cm high). One of the short sides was made of an acrylic sheet and contained an escape tube of black plastic (diameter, 4 cm, length, 4 cm), with its lower edge at 3 cm above the base, at mouse head height. The escape pipe was fitted onto the escape tube. The floor of the black corridor was filled with water (18° ± 1°C) to a depth of 2 cm. To begin the black corridor procedure, each animal was placed in the far end furthest from the exit, to learn the principle of escaping into the tube. The mice received four trials in 1 day, with each trial lasting a maximum of 30 s. If an animal did not escape by the cut-off time, it was gently guided to the exit.

On day 2, mice experienced the clear corridor (Figure 1A), which was located inside the clockmaze. The purpose of this stage was to continue the familiarization to the procedural aspects of the task, while also allowing the animals to see the distal cues in the testing area. The clear corridor was made of three walls (clear plastic, 38 cm long, 9 cm wide, 39 cm high) and an open side that was placed facing a single open tunnel of the clockmaze. The escape pipe was fitted onto the tunnel. The floor of the clockmaze was filled with water (18° ± 1°C) to a depth of 2 cm. Mice received four trials in the clear corridor. Each subject was placed at the end opposite to the exit and was given 30 s to escape into the tube. Animals that did not escape by the cut-off time were guided to the exit.

### Behavioral Testing

For the task proper, 11 escape holes in the clockmaze were sealed while one hole was open and led to the dry open pipe (the “true exit”). A trial in the PPT consisted of placing a mouse at the center of the clockmaze and allowing it to escape into the open tube and connected pipe. The subject was given 60 s to find the true exit and was gently guided to it if it exceeded the cut-off time. The pipe was then removed and the mouse was transported to the drying station (with an infrared lamp providing heat from above) in which it stayed for 3–5 min before being returned to the home cage. Before the next trial, feces were removed and the water was gently stirred to avoid the presence of location-specific olfactory cues. The water temperature was monitored constantly and, if necessary, ice was added to keep it cold (18° ± 1°C). We found in our pilot experiments that holding the water at the target temperature was essential to trigger an immediate escape response in the animals, and when the water temperature was higher (i.e., over 20°C) the animals remained immobile in the center of the pool for extended periods.

The path traversed by the animal, the time to find the true exit and the number of visits to decoys were recorded by Ethovision software (Figure 1B, Supplementary Figure S1B). An error was defined as a frontal or side inspection of a decoy, in which half a body length including the nose was inside the decoy zone, defined as a circular area (radius, 2 cm). Mice received four trials per day, with an inter-trial interval of 15–20 min, for a total of 12 trials.

**Figure 2:**
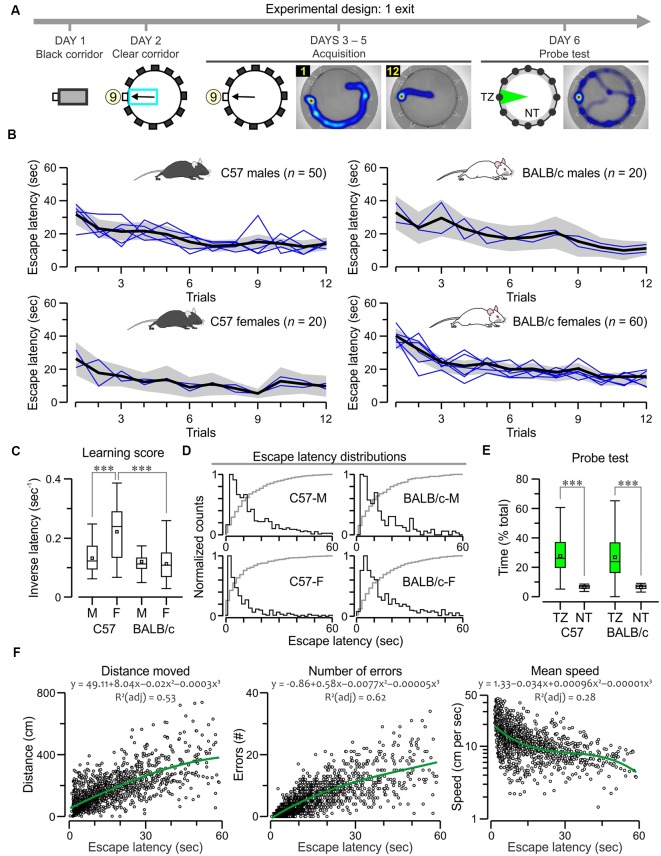
C57 and BALB/c mice show comparable acquisition of the 1-exit test. **(A)** Experimental design of the task, showing plan views of the apparatus with hole 9 as the true exit and heatmaps for a subject on trials 1, 12 and the probe test. Abbreviations: TZ, target zone; NT, non-target zones. **(B)** Plots of escape latency across trials for C57 and BALB/c groups. The black lines denote the ensemble means for the time series, the gray areas are the 5%–95% range, and the blue lines are means for the each separately tested cohort (*n* = 10). Both strains learn the task comparably; *F* > 3, *P* < 0.0001, repeated measures analysis of variance (RMANOVA; exact values in “*Results*” section). **(C)** Boxplot of learning scores (i.e., inversed latencies, which are calculated by taking the values for all trials for each animal) showing that C57 females have the highest scores (***P < 0.0001, *t*-test) and the other groups have similar scores. **(D)** Histograms of escape latencies (black lines) for successful trials that lasted less than 60 s. The gray lines are cumulative probabilities. **(E)** Boxplot of search time in the TZ (% of total time, 60 s) and the NT (expressed as the average of the 11 decoys) for BALB/c (*n* = 30) and C57 (*n* = 20) groups, both of which show significantly higher TZ exploration (***P < 0.0001, *t*-test). **(F)** Scatterplots showing a correlation of escape latency (*n* = 1,597 trials that lasted less than 60 s) with distance moved, number of errors and mean speed (plotted in log scale). Green lines represent polynomial fits for the datasets, which are described by the corresponding functions.

The “one-exit test” (Figures 1, 2A), sometimes referred to as a reference memory test, consisted in training the animals to find the same true exit across 12 trials (four trials per day, with an inter-trial interval of 15–20 min). The day after training, mice were subjected to a single “probe test” in which they were placed in the middle of the clockmaze and stayed in the apparatus for 60 s with all the exits blocked.

**Figure 3:**
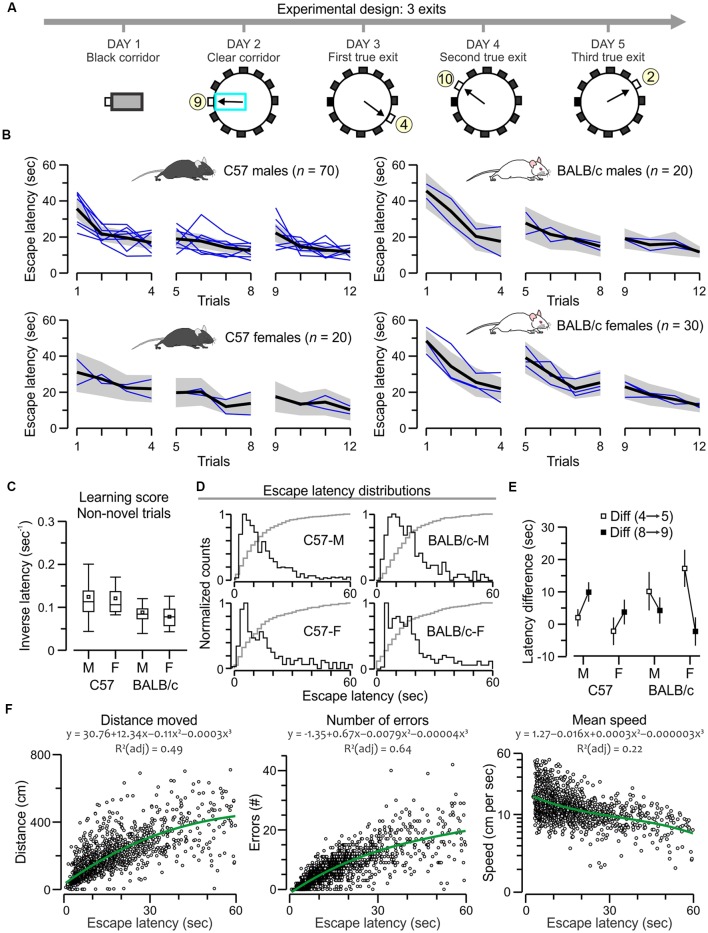
C57 and BALB/c mice show comparable acquisition of the many-exits test.** (A)** Experimental design of the task, showing plan views of the apparatus with a different true exit for each acquisition day. **(B)** Plots of escape latency across trials for C57 and BALB/c groups. The black lines denote the ensemble means for the time series, the gray areas are the 5%–95% range, and the blue lines are means for the each separately tested cohort (*n* = 10). Both strains learn the task comparably; *F* > 3, *P* < 0.0001, RMANOVA (exact values in “*Results*” section). **(C)** Boxplot of learning scores for non-novel trials (2, 3, 4, 6, 7, 8, 10, 11, 12) showing similar scores for all groups (*P* > 0.05 for all *t*-tests). **(D)** Histograms of escape latencies (black lines) for successful trials that lasted less than 60 s. The gray lines are cumulative probabilities. **(E)** Plot of latency difference between trials with a new exit vs. the immediately previous trials (5–4, 9–8) showing positive values for all transitions, suggesting that all mice react to a novel exit. Notably, C57 groups increase the difference values from the first to the second transition (strong novelty effect), whereas BALB/c mice decrease the difference values suggesting a different approach to novelty in this task. **(F)** Scatterplots showing a correlation of escape latency (*n* = 1,513 trials that lasted less than 60 s) with distance moved, number of errors and mean speed (plotted in log scale). Green lines represent polynomial fits for the datasets, which are described by the corresponding functions.

For the “many-exits test,” animals had to find different exit locations that were switched on a daily basis with a pseudo-random scheme. Thus, an animal needed to learn a new exit on a daily basis. In this study, we implemented the many-exits protocol by using three exits (Figure 3A) and eight exits (Figure 6C). In the 3-exits version, a mouse was trained to escape, for example, through exit 4 on the 1st day (trials 1–4), exit 10 on the 2nd day (trials 5–8), and exit 2 on the last day (trials 9–12), so the true exit was moved three times and the mouse had to learn three escape routes. In the 8-exits version, animals received five trials per day on days 1–4 and four trials per day on days 5–8, so for instance, a mouse was trained to escape through exit 7 on day 1 (trials 1–5), *via* exit 11 on day 2 (trials 6–10), *via* exit 4 on day 3 (trials 11–15), *via* exit 9 on day 4 (trials 16–20), *via* exit 3 on day 5 (trials 21–24), *via* exit 5 on day 6 (trials 25–28), *via* exit 1 on day 7 (trials 29–32), and through exit 6 on day 8 (trials 33–36).

**Figure 4:**
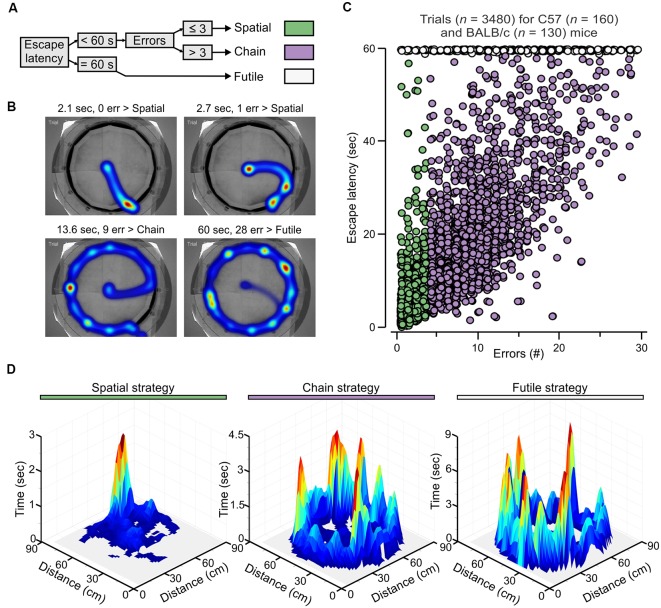
Escape strategy analysis of the PPT.** (A)** Escape strategy classification algorithm based on escape latency and the number of errors. **(B)** Representative heatmaps for mice escaping to the true exit highlight the use of different strategies. Abbreviation: err, errors. **(C)** Graph of the number of errors vs. escape latency for individual trials of fully trained mice (*n* = 290) showing segregation of strategies into three clusters (colors as in **A**). **(D)** Three-dimensional surface plot showing the summation of the occupancy times taken from 40 randomly chosen trials, from different subjects with the same true exit (hole 11), for each escape strategy.

**Figure 5:**
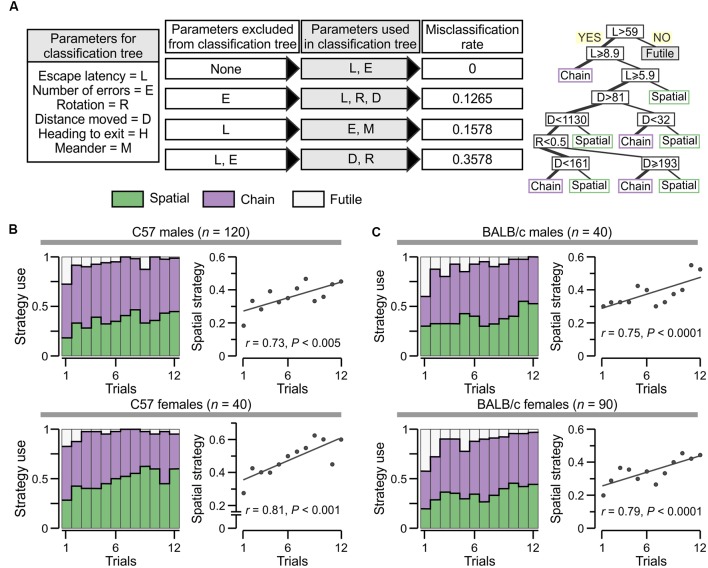
Escape strategy analysis applied to the groups tested in the PPT. **(A)** *Left*, parameters used in classification and regression tree (CART) models to determine escape strategy predictors: rotation = number of full rotations done by an animal; distance moved = space (cm) covered; heading to exit = angle (degrees) to which an animal moves toward the true exit; and meander = number of direction changes done by an animal. *Middle*, Exclusion of certain parameters in the CART models and resulting misclassification rate for each case. As expected, including all parameters gives null misclassification error, whereas excluding escape latency from any CART results in an inability to predict the futile strategy. *Right*, CART model in which the number of errors is excluded can predict the three strategies with a misclassification score of 12.65%. **(B)** Escape strategy analysis, with colors as in Panel **(A)**, applied to C57 males (*top*) and females (*bottom*). The plots (at *right*) represent the use of spatial strategy across trials with a linear fit for the data. **(C)** Escape strategy analysis of BALB/c males (*top*) and females (*bottom*). Scatterplots (at *right*) for the use of spatial strategy across trials with a linear fit for the data.

**Figure 6:**
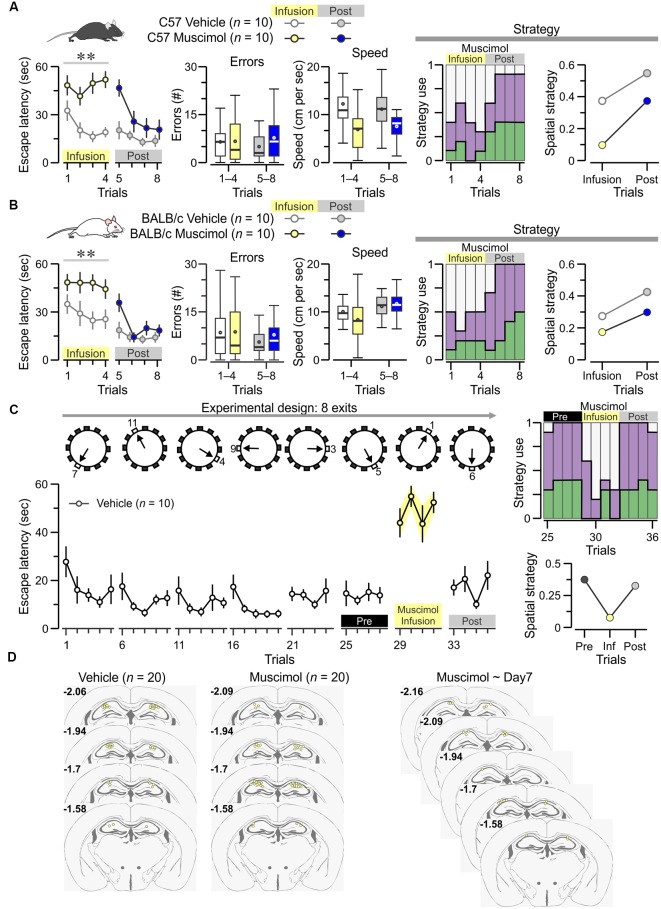
Dorsal hippocampal CA1 region is required for acquisition of the PPT. **(A)** *Left*, plot of escape latency [mean ± standard error of the mean (SEM)] across trials for C57 mice (*n* = 10 females) receiving bilateral injections of muscimol on trials 1–4 and vehicle injections on trials 5–8. Vehicle-treated animals (*n* = 10) are used as controls. On trials 1–4, the muscimol group is unable to acquire the task (*F*_(1,3)_ = 26.7, **P = 7.7 × 10^−5^ RMANOVA of log_10_ transformed escape latencies). *Middle*, boxplots of the number of errors and the mean speed demonstrate no significant effect of muscimol. *Right*, Escape strategy analysis of the muscimol-infused group (colors as in Figure 4A) show marked use of futile strategy on trials 1–4 (muscimol) and significant increase of spatial strategy on trials 5–8 (post-muscimol). **(B)** A similar study of BALB/c females (10 muscimol, 10 vehicle) with plot of escape latency (*left*) showing that, on trials 1–4, the muscimol-infused mice do not acquire the task (*F*_(1,3)_ = 10.98, **P = 0.0038, RMANOVA of log_10_ transformed escape latencies) but have a normal number of errors and mean speed (*middle*). Escape strategy analysis (*right*) of the muscimol-infused group shows they mostly use a futile strategy on trials 1–4 (muscimol) and increase their use of spatial strategy on trials 5–8 (post-muscimol). **(C)** *Top*, diagram of the many-exits test, in which the true exit is switched on a daily basis, and animals are tested on eight different exits. *Bottom*, plot of escape latency (mean ± SEM) across trials for vehicle-treatedanimals (*n* = 10). As indicated by the labels, mice are treated with muscimol on day 7 (trials 29–32), which produces significant performance impairment (*T*_(9)_ = 8.7, *P* = 1.1 × 10^−5^, paired *t*-test for four trials immediately before muscimol vs. muscimol trials). **(D)** Cannula locations for the groups described above. Numbers indicate anterior-posterior coordinates, according to Paxinos and Franklin (2012).

### Escape Strategy Analysis

A classifier based on escape latency and the number of errors (blocked exits visited) was developed to distinguish escape strategies (Figure 4). Over the course of testing, naive animals displayed a shift of their movement pattern in the PPT from examining several consecutive blocked exits before escaping through the true exit (>3 errors) towards a directed escape strategy. In the latter case, animals directly entered the target quadrant and escaped the maze with ≤3 errors. The subset of animals that failed to escape the PPT within 60 s was classified as having used a futile escape strategy. The parameters were quantified by software (Ethovision), although we performed a pilot study in which human observers validated the error counts using printed track plots and video replay. Escape strategies were computed in Excel (Microsoft). Moreover, the analysis was validated by applying “classification and regression tree” (CART) predictive modeling, which was used to assess the impact of quantifiable parameters (listed and explained in Figure 5A) on solving strategies, and also for constructing decision trees. For CART data analysis, model fitting and plotting we used the R packages called *tree*, *caret*, and *ggplot* (Wickham, 2009; Kuhn et al., 2016; Ripley, 2016).

### Dorsal Intra-hippocampal Injections

C57 (10 males, 10 females) and BALB/c (10 males, 10 females) mice were used for this assay. Animals were induced (isoflurane at 3.5% with a vaporizer (Tec 3 Leading Edge, Centennial, CO, USA) and maintained (isoflurane at 1.5%–2%) in the supine position. They were fixed in a stereotaxic frame (Kopf) and implanted with a double guide cannula (26-gauge, Plastic One, Roanoke, VA, USA), which ended intra-hippocampally. Stereotaxic coordinates were chosen based on Paxinos and Franklin (2012): AP, −2 mm; ML, 1.5 mm; DV, 1.4 mm. Cannulas were fixed to the skull using dental acrylic (Stoelting). After the implant, animals recovered for 1 week. On the day of infusion, bilateral injection needles were inserted into the guide cannulas, with the needles extending 1 mm beyond the cannula tip. Mice were bilaterally infused with vehicle, which consisted of artificial cerebrospinal fluid (ACSF; Chang et al., 2006), or muscimol at 2.6 nmol (1 μg/μL; 8.74 mM, Sigma Aldrich, St. Louis, MO, USA). A microdialysis pump (PHD 2000, Harvard Apparatus, Holliston, MA, USA) fitted with Hamilton microliter syringes, delivered the drugs at a flow rate of 0.25 μL per min (for 2 min) to attain a final volume of 0.5 μL per hemisphere. The needles were left in place for 1 min, they were then removed, dummy cannula tops were re-inserted, and mice were left in a holding cage in the experimental room for 30–45 min before behavioral assessment. Upon experiment completion, correct cannula placement was verified *post mortem*.

### Comparison Between the PPT and the MWM

**Figure 7:**
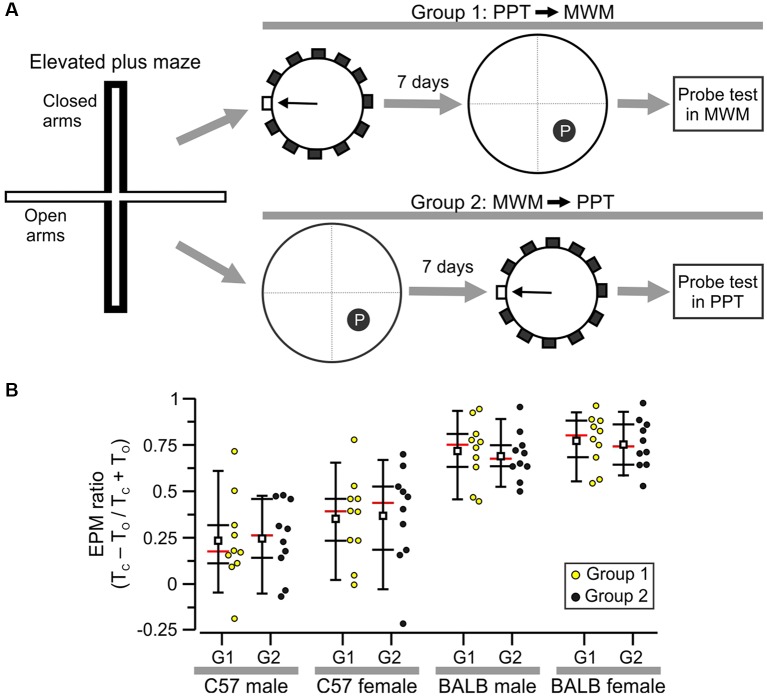
Selection of subjects for a comparison of the PPT and Morris water maze (MWM). **(A)** Experimental design showing that all mice were first tested in the elevated plus maze (EPM) task and were then assigned to Group 1 (PPT followed by MWM) or Group 2 (MWM followed by PPT). **(B)** Plot of EPM ratios with each circle representing the value of an individual subject. These values were used to separate animals into Groups 1 and 2 for each sex/strain cohort. Abbreviations: T_C_, time in closed arms; T_O_, time in open arms.

C57 mice (20 males, 20 females) and BALB/c mice (20 males, 20 females) were used in a counterbalanced design to assess their performance in the PPT and MWM. The same experimenters conducted both tasks at the same time of the day, and the mazes were positioned in the same spot in the testing area. Each strain/sex (*n* = 20) cohort was divided into Group 1 (*n* = 10), which was first tested in the PPT and 7 days later in the MWM, and Group 2 (*n* = 10) that was first assessed in the MWM and 7 days later in the PPT (Figure 7A). The same number of trials (*n* = 12) was used in each of the two tasks. To assign the mice to either Group 1 or 2, the animals were subjected to the elevated plus maze (EPM) task, and the EPM ratios (see below) for the individuals were organized so that Groups 1 and 2 had matching mean EPM ratios and *Z* scores.

Our protocol to implement the MWM task has been described extensively (Tsien et al., 1996; Chang et al., 2006, 2009; Vingtdeux et al., 2016). Briefly, we implemented the hidden platform variant of the MWM with the use of a circular pool (diameter, 160 cm) filled with clear water (21° ± 1°C). The experimental area, including the distal cues, used in the MWM was exactly the same as in the PPT. We trained the animals in blocks of four trials per day for a total of 12 trials (3 days). The inter-trial interval was ~20 min. A mouse was lowered into the pool at one of four starting points in the periphery and had to swim and climb to a hidden platform (diameter, 16 cm), located at the center of the southeast quadrant, in less than 60 s (cut-off time). Subjects who failed a trial were guided to the platform and stayed on it for ~5 s. The paths traversed by the animals and the times to find the platform were recorded by Ethovision software.

### EPM Task

The apparatus consisted of two open arms (35 × 5 cm) crossed with two closed arms (35 × 5 × 20 cm) made from acrylic (painted gray). The EPM was elevated to a height of 1 m and two procedure lights (model GS900, Welch Allyn, Skaneateles Falls, NY, USA) were placed 1 m above it to provide ~200 lux. An overhead digital camera, fixed above the EPM, sent the video signal to the tracking Ethovision software. Mice were taken from the home cage, were placed individually on the EPM facing an open arm, and spent 5 min exploring it. The software calculated the cumulative time in the closed arms (T_C_) and the cumulative time in the open arms (T_O_), which were used to compute the EPM ratio defined as (T_C_ − T_O_)/(T_C_ + T_O_).

### Rotarod Task

The apparatus was a five-lane rotating rod (model ENV-574M, Med Associates, St. Albans, VT, USA). Mice underwent an accelerating protocol (4–40 rpm) in which they were placed in the horizontal rod and were allowed to stay on it until the cut-off time (300 s). There were three trials per animal, with an inter-trial interval of ~15 min. The riding time for each trial was recorded by software (Rota-Rod 2, Med Associates) and used for statistical comparisons.

### Determination of Serum Corticosterone (CORT) Levels

For this procedure, blood samples (~0.5 mL) were collected from the facial vein, kept at room temperature (30 min), and then centrifuged (6,000 rpm, 10 min, 4°C), the supernatants were collected and re-centrifuged (6,000 rpm, 10 min, 4°C), and the sera were collected and stored (−80°C). CORT ELISA kit (Enzo Life Sciences, Farmingdale, NY, USA, Cat #ADI-901-097) was used for CORT quantification, following the manufacturer’s specifications. To avoid inter-assay variance, we analyzed all the samples in a single run.

### Measurement of Body Temperature

During the counterbalanced study of the PPT and MWM, the body temperature of mice was measured immediately before and after each trial, with the use of a handheld thermography camera (FLIR E6, FLIR Systems, Wilsonville, OR, USA), allowing for an almost instantaneous and noninvasive readout. The camera was factory-calibrated, with 320 × 240 pixels (9 Hz refresh rate) and a built-in screen for instant visual focus control. Built-in thermal optics permitted to focus from ~0.5 m to infinite automatically. The pixels that included the top of the head and the eyes were used to determine the animal’s temperature, as these areas have been shown to correlate well with the core temperature of mice (Vogel et al., 2016).

### Statistical Analysis

Parametric and non-parametric statistical tests were chosen based on the characteristics of each dataset. Tests were computed with statistical packages in Origin Pro (version 9.1, OriginLab) and R (R project for statistical computing^1^). Tests are indicated in the main text for each comparison along with the appropriate statistic and the exact *P* value. A value of *P* < 0.05 was considered significant.

## Results

### Analysis of Escape Latency in the PPT

We implemented the PPT with slight modifications from the original report by Deacon and Rawlins (2002). Each set was subjected to a timeline (Figure 1A) of pre-training in a black corridor (day 1, 4 trials) and a clear corridor (day 2, 4 trials), followed by PPT training (days 3–5, 12 trials in total with four trials per day). The apparatus consisted of a clear cylinder with 12 circular holes in its wall, arranged like the face of a clock, and filled with water to a depth of 2 cm (Figure 1A, Supplementary Figure S1A). The holes were 2.5 cm above the floor and were sealed with black plugs, except for a single hole that was open and connected to a black pipe (called true exit henceforth). Animals were gently placed at the center of the arena and waded through the water, using distal cues to guide navigation, to find the true exit while avoiding the 11 decoys. The cut-off time for a trial was 60 s.

For the one-exit test, we assessed the escape latencies (defined as the time taken to find the true exit) of C57 mice (50 males, 20 females) and BALB/c mice (20 males, 60 females). We found they all acquired the task with ease as they decreased their escape latencies across trials to a stable value (Figures 1B, 2B). A repeated measures analysis of variance (RMANOVA) of escape latency (log_10_ transformed to satisfy the requirements of equal variance and normality), with trials as the repeated measure, showed a significant effect for C57 males (*F*_(1,11)_ = 5.7, *P* = 9.7 × 10^−9^), C57 females (*F*_(1,11)_ = 4.3, *P* = 7.8 × 10^−6^), BALB/c males (*F*_(1,11)_ = 3.6, *P* = 9.6 × 10^−5^) and BALB/c females (*F*_(1,11)_ = 13.4, *P* = 2.3 × 10^−23^). We used learning scores (i.e., inversed latency) to compare the groups (2C) and found that C57 females had the highest scores (C57 females vs. C57 males, *T*_(68)_ = 5.4, *P* = 9.4 × 10^−7^; C57 females vs. BALB/c females, *T*_(78)_ = 6.2, *P* = 2 × 10^−8^, *t*-test) while the other groups had comparable learning scores. Moreover, analysis of escape latency distributions confirmed that C57 females had shorter escape latencies than the other groups (Figure 2D). A probe test in which the true exit was blocked revealed significantly higher exploration of the target area, near the exit, in comparison to the decoys (Figure 2E; C57, *T*_(18)_ = 5.9, *P* = 1.0 × 10^−5^; BALB/c, *T*_(28)_ = 6.1, *P* = 1.3 × 10^−6^; paired *t*-test), but no difference between strains (*T* = 0.16, *P* = 0.87, *t*-test). Thus, BALB/c and C57 mice displayed strong acquisition and retention of the one-exit test in the clockmaze.

Escape latency is commonly recorded as the readout of the MWM and other spatial learning tasks; therefore, we decided to analyze it as the independent variable to other parameters in the PPT. In an effort to avoid spurious correlations, we studied only successful trials (*n* = 1,597) that mice completed before the cut-off time in the one-exit test. Figure 2F shows that the escape latency was positively correlated to the distance moved (*ρ* = 0.72, *P* < 0.0001, Spearman correlation) and the number of errors defined as blocked decoys visited prior to the true exit (*ρ* = 0.82, *P* < 0.0001) but not the mean speed (*ρ* = −0.55, *P* < 0.0001).

For the many-exits test, animals needed to escape to a different exit on a daily basis (Figure 3A). We implemented a 3-exits version and evaluated the escape latencies of C57 mice (70 males, 20 females) and BALB/c mice (20 males, 30 females). All mice acquired the task rapidly as their escape latencies decreased across trials (Figure 3B) with statistical significance (C57 males, *F*_(1,11)_ = 10.7, *P* = 1.8 × 10^−18^; C57 females, *F*_(1,11)_ = 3.3, *P* = 3.9 × 10^−4^; BALB/c males, *F*_(1,11)_ = 6.8, *P* = 1.1 × 10^−9^; BALB/c females, *F*_(1,11)_ = 10.3, *P* = 4.7 × 10^−16^, RMANOVA of log_10_ transformed escape latencies). We computed the inversed latency, excluding the novel trials in which the mice faced a new exit (trials 1, 5, and 9), and found comparable learning scores between groups (Figure 3C; *P* > 0.05 for all *t*-tests), a result that was corroborated by the escape latency distributions that were also similar between groups (Figure 3C). We also examined the animal’s reaction to a novel exit, by calculating the latency difference between a trial with a new exit vs. the previous trial (trials 5 minus 4, and trials 9 minus 8); C57 mice showed increasing difference values suggesting they were confounded by the switch and had to readjust their approach, whereas BALB/c mice showed decreasing difference values suggesting a different approach to novelty in this task (Figure 3D). Additionally, we examined the correlations between escape latency and other parameters in the successful trials (*n* = 1,513) of the many-exits test, and found that escape latency was positively correlated to the distance moved (*ρ* = 0.77, *P* < 0.0001, Spearman correlation) and the number of errors (*ρ* = 0.83, *P* < 0.0001) but not the mean speed (*ρ* = −0.43, *P* < 0.0001).

### Escape Strategy Analysis of the PPT

In human subjects, hippocampus-based learning facilitates problem solving through associative pairing of previously unrelated cues (Gold et al., 2006). In the clockmaze, animals need to pair the location of the true exit with the position of distal cues outside the maze. Therefore, in analogy with humans, learning within the clockmaze would be facilitated by a behavioral change away from exploratory sampling of closed decoys and towards directed movement to the true exit. We reasoned that such behavioral change could be quantified with a strategy algorithm based on escape latency and the number of errors (Figures 4A,B). Escape latency was used as the categorical variable to distinguish those trials in which animals failed to find the exit within the cut-off time (60 s, which we labeled futile strategy (Figure 4A), from trials in which they were successful (escape latency <60 s). Subsequently, the number of errors determined the assignment to a spatial strategy (errors ≤3) or a chain strategy (errors >3; Figure 4A). We then applied this analysis to all mice (*n* = 290) tested in the one-exit test (Figure 2) and many-exits test (Figure 3). By plotting the number of errors vs. escape latency for individual trials (*n* = 3,480), we were able to segregate the escape strategies into three clusters, with the whole ensemble representing a two-dimensional strategic matrix (Figure 4C). To further demonstrate that the escape strategy analysis segregated distinct navigational paths, we used the tracking data (obtained at 30 samples per second) to create surface plots of selected trials classified as spatial, chain and futile (*n* = 40 trials for each plot) and found that the navigational paths were indeed distinctive for each escape strategy (Figure 4D). Interestingly, for spatial trials the mice showed a strong spatial bias, seen as a single peak around the true exit; for chain trials the mice made stops in many decoy exits until they found the true exit, seen as several peaks with the highest around the true exit; and for futile trials the navigational pattern resembled the chain trials, with visits to all the decoy exits but without a high peak around the true exit (Figure 4D).

To further test the usefulness of the escape latency and the number of errors as strong escape strategy predictors, we applied CART models (Wickham, 2009; Kuhn et al., 2016; Ripley, 2016), using descriptive parameters that are also commonly recorded in the MWM and the Barnes maze (Figure 5A). We studied the rate of escape strategy misclassification when a given parameter was omitted in a CART model and found that exclusion of the number of errors yielded a misclassification score of 12.7%, omission of the escape latency gave a misclassification of 15.8%, whereas exclusion of both the escape latency and the number of errors increased the misclassification to 35.8% (Figure 5A). Furthermore, when the escape latency was omitted in any of the CART models, it was not possible to predict the futile strategy.

We applied the strategy algorithm in a trial-by-trial basis during PPT acquisition by C57 mice (Figure 5B; males, *ρ* = 0.73, *P* < 0.005; females, *ρ* = 0.81, *P* < 0.005, Spearman correlation) as well as BALB/c mice (Figure 5C; males, *ρ* = 0.68, *P* < 0.05; females, *ρ* = 0.73, *P* < 0.005). Animals from both strains clearly displayed strategic flexibility, that is, they increased their use of spatial strategy as the test progressed. Additionally, the futile strategy was negligible by the end of the task.

### The PPT Requires the Dorsal CA1 Region of the Hippocampus

We sought to study whether pharmacological inactivation of the dorsal CA1 region, attained by administration of the GABA_A_ receptor agonist muscimol (Moser and Moser, 1998) into bilaterally cannulated mice, would lead to impaired performance. On day 1, pre-trained C57 females that received muscimol, 30 min before training, were unable to acquire the PPT (Figure 6A *left*; *n* = 10, *F*_(1,3)_ = 26.7, *P* = 7.7× 10^−5^ RMANOVA of log_10_ transformed escape latencies) whereas vehicle-injected C57 mice acquired the task efficiently. The very high values for escape latency in the muscimol-treated mice suggest that the drug may have also interfered with the familiarization aspects of the task, such as navigating in the cold water (instead of simply staying immobile) and entering into the open hole and the adjacent pipe as a means of escape. On day 2, all animals were vehicle-injected, and the mice that had received muscimol (on day 1) displayed lower escape latencies (Figure 6A *left*; *F*_(1,3)_ = 4.93, *P* = 0.007, RMANOVA of log_10_ transformed escape latencies), which were significantly different from those during muscimol (*T*_(9)_ = 3.1, *P* = 0.01, paired *t*-test). Of note, muscimol did not affect significantly the number of errors nor the mean speed (Figure 6A, *middle*). Analysis of the escape strategies employed during the task confirmed that, on day 1, the muscimol-infused mice did not rely on spatial strategy but used a futile strategy instead; however, on day 2 (post-infusion), these same mice showed improved use of spatial strategy (Figure 6A, *right*).

We repeated the muscimol study in BALB/c mice and found that, on day 1, pre-trained females receiving muscimol did not acquire the PPT (Figure 6B *left*; *n* = 10, *F*_(1,3)_ = 10.98, *P* = 0.0038, RMANOVA of log_10_ transformed escape latencies) whereas, on day 2, the same animals showed decreased escape latencies (Figure 6B *left*; *F*_(1,3)_ = 3.7, *P* = 0.002, RMANOVA), which were significantly lower to those on day 1 (*T*_(9)_ = 6.2, *P* = 1.7 × 10^−4^, paired *t*-test). Escape strategy analysis for the task demonstrated that, on day 1, the muscimol-infused mice used spatial strategy marginally but, on day 2 (post-infusion), the same mice increased their use of spatial strategy (Figure 6B, *right*).

Next, we asked whether the dorsal CA1 region was required for flexible retrieval of the true exit location at a late stage in training. To this end, we used the many-exits test in which vehicle-injected mice (*n* = 10, bilaterally cannulated BALB/c females) were trained to find a different true exit each day (Figure 6C; 36 trials distributed across 8 days). On day 7, muscimol was administered instead of vehicle and the mice showed impaired performance (Figure 6C; *T*_(9)_ = 8.7, *P* = 1.1 × 10^−5^, paired *t*-test for four trials immediately before muscimol vs. muscimol trials), which was restored after a washout period of 24 h (Figure 6C; *T*_(9)_ = 7.5, *P* = 3.5 × 10^−5^, paired *t*-test for muscimol vs. post-muscimol trials). Escape strategy analysis revealed that, during the muscimol trials, there was a negligible use of spatial strategy and strong incidence of futile strategy; the use of spatial strategy was reinstated on the post-muscimol trials (Figure 6C, *right*). After testing was ended, the animals were sacrificed and the cannula locations were confirmed to have reached dorsal CA1 (Figure 6D). Thus, we have demonstrated that the active involvement of the dorsal CA1 region is required for the acquisition of spatial locations at the early stages as well as at the late stages of the PPT in the clockmaze.

### Comparison Between the PPT and the MWM

To examine whether mouse strain can affect the performance in the PPT and MWM, we studied BALB/c mice (20 males, 20 females) and C57 mice (20 males, 20 females) with a counterbalanced design. Group 1 was first tested in the PPT and 7 days later in the MWM, and Group 2 was assessed in the MWM first and the PPT afterward (Figure 7A). Moreover, all animals were tested beforehand in the elevated plus maze task and their individual EPM ratios were used to assign subjects to either Group 1 or 2 so that the means and *Z* scores were matched for each strain/sex combination (Figure 7B). The values were: C57 males, Group 1, mean = 0.233 ± 0.244; Group 2, mean = 0.247 ± 0.197; C57 females, Group 1, mean = 0.353 ± 0.232; Group 2, mean = 0.369 ± 0.269; BALB/c males, Group 1, mean = 0.718 ± 0.167; Group 2, mean = 0.692 ± 0.132; BALB/c females, Group 1, mean = 0.772 ± 0.139; Group 2, mean = 0.756 ± 0.135 (all values are mean ± SD). Notably, BALB/c animals showed higher anxiety as evidenced by their significantly higher EPM scores (Figure 7B; C57, *n* = 40, mean = 0.3 ± 0.236; BALB/c, *n* = 40, mean = 0.735 ± 0.142, *T*_(78)_ = 9.9, *P* = 1.4 × 10^−15^, *t*-test). We implemented the hidden platform variant of the MWM (Tsien et al., 1996; Chang et al., 2006, 2009; Vingtdeux et al., 2016), with the use of a circular pool (diameter, 160 cm) in which we trained animals in blocks of four trials per day. A mouse was lowered into the pool at one of four starting points in the periphery and had to swim and climb to a hidden platform in less than 60 s (cut-off time). Subjects who failed a trial were guided to the platform and stayed on it for ~5 s. We decided to use escape latency as the dependent variable to compare the performance of mice in the PPT and the MWM, as this is a widely accepted metric for the latter.

**Figure 8:**
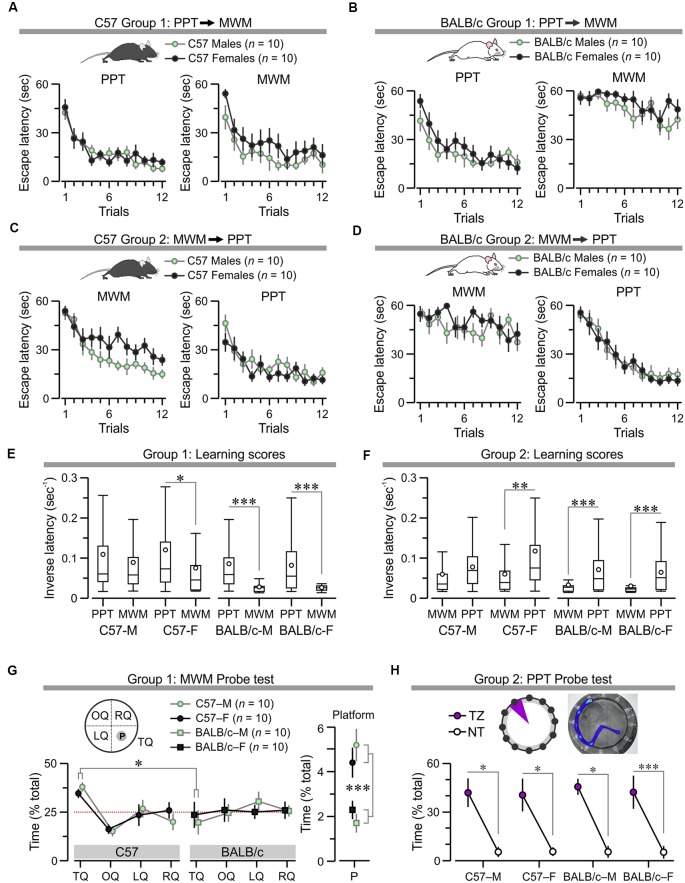
Comparison of the PPT and MWM in C57 and BALB/c mice. **(A–D)** Plots of escape latency (mean ± SEM) across trials, arranged by group/strain, showing that C57 mice acquire the PPT and the MWM normally and independently of testing order **(A,C)**, while BALB/c mice acquire the PPT normally but are impaired in the MWM **(B,D)**. **(E)** Boxplot of learning scores (i.e., inversed latency calculated for all trials) for subjects in Group 1 shows that only C57 males (C57-M) learn the PPT and the subsequent MWM with equal ease (*T*_(238)_ = 1.35, *P* = 0.18, *t*-test), whereas the other groups show poorer acquisition of the MWM (C57 females, C57-F, *T*_(238)_ = 2.79, *P = 0.004; BALB/c males, BALB/c-M, *T*_(238)_ = 7.17, ***P = 9.48 × 10^−12^; BALB/c females, BALB/c-F, *T*_(238)_ = 8.41, ***P = 3.74 × 10^−15^, *t*-test).** (F)** Boxplot of learning scores for subjects in Group 2 shows that C57 males learn the MWM and the PPT equally (C57-M, *T*_(238)_ = 2.34, *P* = 0.02, *t*-test), whereas the other cohorts learn the MWM more poorly than the PPT (C57-F, *T*_(238)_ = 3.93, **P = 0.0001; BALB/c-M, *T*_(238)_ = 5.72, ***P = 3.26 × 10^−8^; BALB/c-F, *T*_(238)_ = 6.53, ***P = 4.1 × 10^−10^, *t*-test). **(G)** Subjects in Group 1 were subjected to a probe test following the MWM. The left plot shows preferential search of the target quadrant for C57 mice but not BALB/c mice (*T*_(38)_ = 3.13, *P = 0.003, *t*-test). The right plot shows that C57 mice spend significantly more time in the platform location than BALB/c mice (*T*_(38)_ = 4.89, *P* = 1.89 × 10^−5^, *t*-test). **(H)** Subjects in Group 2 experienced a probe test in the clockmaze. *Top*, representative heatmap of a subject who had been trained to escape through exit 11. Abbreviations: TZ, target zone; NT, non-target zones. *Bottom*, plot showing preferential search of the TZ in all groups (C57-M, *T*_(18)_ = 4.12, *P = 6.5 × 10^−4^; C57-F, *T*_(18)_ = 3.43, *P = 0.002; BALB/c-M, *T*_(18)_ = 3.97, *P = 8.97 × 10^−4^; BALB/c-F, *T*_(18)_ = 7.2, ***P = 1.06 × 10^−6^, *t*-test).

The counterbalanced study revealed that C57 males and females acquired both the PPT and the MWM regardless of the order of testing (Figures 8A,C). Conversely, BALB/c males and females learned the PPT normally but were impaired in finding the location of the hidden platform in the MWM (Figures 8B,D). Statistical analysis of Group 1 showed no difference for C57 males and females (Figure 8A; PPT, *F*_(1,11)_ = 0.32, *P* = 0.58; MWM, *F*_(1,11)_ = 2.22, *P* = 0.15; two-way RMANOVA of log_10_ transformed escape latencies) and also for BALB/c males and females (Figure 8B), PPT, *F*_(1,11)_ = 0.22, *P* = 0.65; MWM, *F*_(1,11)_ = 0.98, *P* = 0.34). For animals in Group 2, males and females of each strain showed comparable performance (Figure 8C), C57: PPT, *F*_(1,11)_ = 5.04, *P* = 0.038; (Figure 8D), BALB/c: MWM, *F*_(1,11)_ = 0.96, *P* = 0.34; PPT, *F*_(1,11)_ = 0.013, *P* = 0.9), with the exception of C57 mice in the MWM, for which males were significantly faster (Figure 8C; *F*_(1,11)_ = 10.44, *P* = 0.0046). Furthermore, a comparison of learning scores (i.e., inverse latency, calculated for all the trials) showed that each of the subgroups had higher scores in the PPT than the MWM (Figures 8E,F), evidencing that the paddling task demands less effort than the swimming task. Notably, BALB/c mice of either sex had very poor learning scores in the MWM, demonstrating their handicap in learning this task; these mice performed well in the PPT (Figures 8E,F).

Mice in Group 1 were subjected to a probe test in the watermaze 24 h after MWM training. The data revealed that BALB/c males and females did not swim preferentially in the target quadrant, where the platform was located during acquisition, whereas C57 males and females showed robust retention (Figure 8G; BALB/c vs. C57, search time in target quadrant, *T*_(38)_ = 3.13, *P* = 0.003; search time in platform location, *T*_(38)_ = 4.89, *P* = 1.89 × 10^−5^, *t*-test). Furthermore, animals in Group 2 underwent a probe test, 24 h after training in the PPT, which showed that both C57 and BALB/c mice of both sexes strongly preferred the target zone in which the true exit had been during acquisition (Figure 8H; search time in target zone vs. non-target, C57, *T*_(38)_ = 5.44, *P* = 3.39 × 10^−6^; BALB/c, *T*_(38)_ = 7.3, *P* = 9.59 × 10^−9^, *t*-test).

**Figure 9:**
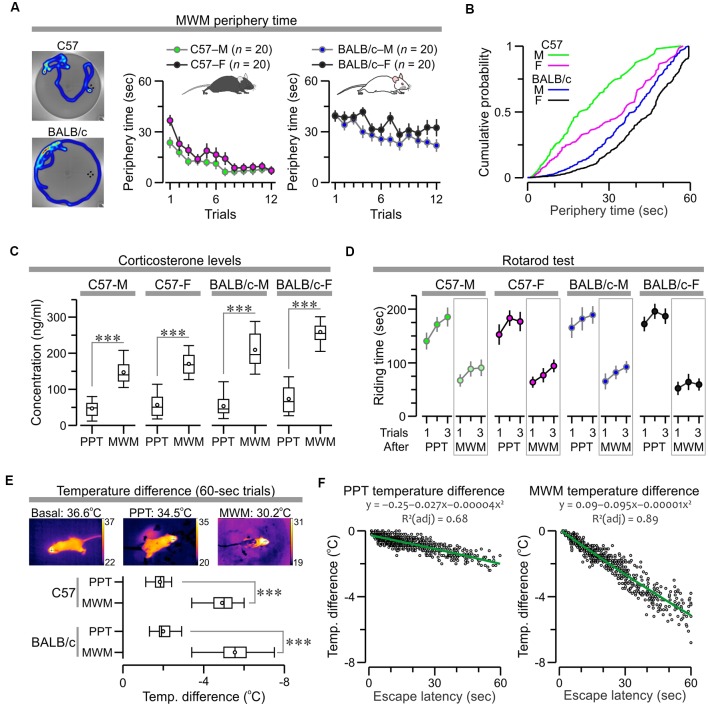
Factors that influence performance in the PPT and MWM. **(A)** *Left*, representative heatmaps of mice swimming in the watermaze. *Right*, plots of periphery time (mean ± SEM) across trials show a clear decrease for C57 mice (C57 males, C57-M, *F*_(1,11)_ = 5.79, *P* = 3.6 × 10^−8^; C57 females, C57-F, *F*_(1,11)_ = 8.19, *P* = 7.1 × 10^−12^, RMANOVA) and elevated values in all trials for BALB/c mice (BALB/c males, BALB/c-M, *F*_(1,11)_ = 4.1, *P* = 1.57 × 10^−5^; BALB/c females, BALB/c-F, *F*_(1,11)_ = 2.68, *P* = 0.003, RMANOVA). **(B)** Probability distributions for periphery time demonstrate a clear thigmotaxis scale, such as BALB/c-F > BALB/c-M > C57-F > C57-M. **(C)** Boxplot of serum corticosterone concentration in mice measured ~15 min after the last trial of the MWM (Group 1) or the PPT (Group 2). MWM values are significantly higher than PPT values (***P < 0.0001, *t*-test; exact values in “*Results*” section). **(D)** Plot of riding time (mean ± SEM) across trials in the rotarod, evaluated ~3 h after the last trial of the MWM (Group 1) or the PPT (Group 2). Mice that had finished the MWM have much shorter riding times as compared to mice that had finished the PPT. **(E)** *Top*, body temperature was monitored with a thermography camera and the pixel values on top of the head were used for analysis. *Bottom*, boxplot of temperature difference in trials that lasted until the cut-off time of 60 s (C57, *T*_(108)_ = 25.74, ***P = 6.8 × 10^−48^; BALB/c, *T*_(393)_ = 38.5, *P* = 1.77 × 10^−35^, *t*-test). **(F)** Scatterplots for all the successful trials (lasting less than 60 s) show an inverse linear correlation between escape latency and temperature difference (PPT, *ρ* = −0.76, *P* < 0.0001; MWM, *ρ* = −0.95, *P* < 0.0001, Spearman correlation). Green lines represent polynomial fits for the datasets, which are described by the corresponding functions.

We examined how factors such as anxiety, exhaustion, and hypothermia could influence performance in the MWM and PPT. The MWM datasets were used to examine thigmotaxis, which is widely considered an index of anxiety in mice (Simon et al., 1994), by calculating the amount of time the subjects spent swimming near the border of the watermaze. C57 mice showed a clear decrease in “periphery time” across trials, whereas BALB/c mice showed elevated periphery time for all trials (Figure 9A; C57 males, *F*_(1,11)_ = 5.79, *P* = 3.6 × 10^−8^; C57 females, *F*_(1,11)_ = 8.19, *P* = 7.1 × 10^−12^; BALB/c males, *F*_(1,11)_ = 4.1, *P* = 1.57 × 10^−5^; BALB/c females, *F*_(1,11)_ = 2.68, *P* = 0.003, RMANOVA). Interestingly, a probability distribution for the periphery times clearly demonstrated a distinctive thigmotaxis level for each subgroup, with BALB/c females being the most anxious subjects, followed by BALB/c males and C57 females, and C57 males being the least anxious animals (Figure 9B).

To obtain another index of stress/anxiety, we measured the serum levels of corticosterone (CORT) in mice, ~15 min after they finished the last trial of the MWM (Group 1) or the PPT (Group 2), and found a dramatic CORT elevation in mice that were just subjected to the MWM, while the CORT levels of mice that had just finished the PPT were comparable to naive mice (BALB/c females, *n* = 5, range = 17–82 ng/mL). Statistical comparisons for each subgroup confirmed these observations (Figure 9C; C57 males, *T*_(18)_ = 7.85, *P* = 3.2 × 10^−7^; C57 females, *T*_(18)_ = 7.94, *P* = 2.7 × 10^−7^; BALB/c males, *T*_(18)_ = 8.34, *P* = 1.36 × 10^−7^; BALB/c females, *T*_(18)_ = 10.37, *P* = 5.1 × 10^−9^, *t*-test).

To evaluate the degree of exhaustion experienced by the animals, we used the rotarod test that was implemented ~3 h after the mice finished the last trial of the MWM (Group 1) or the PPT (Group 2). Mice were placed in an accelerating rod (4–40 rpm) for three trials, with an inter-trial interval of ~15 min, and the riding time for each trial was used for analysis. Mice that had previously finished the MWM had much shorter times to fall from the rod when compared to mice that had finished the PPT, which was confirmed by statistical comparisons for the subgroups (Figure 9D; C57 males, *T*_(58)_ = 7.49, *P* = 4.3 × 10^−10^; C57 females, *T*_(58)_ = 8.11, *P* = 3.97 × 10^−11^; BALB/c males, *T*_(58)_ = 8.23, *P* = 2.53 × 10^−11^; BALB/c females, *T*_(58)_ = 12.64, *P* = 2.6 × 10^−18^, *t*-test).

To study hypothermia induced by exposure to cold water in the MWM and PPT, we measured the body temperature (Figure 9E) with a handheld thermography camera (Vogel et al., 2016), immediately before and after each trial, revealing a temperature drop that was correlated linearly with task duration. Importantly, in the MWM the water was at 21° ± 1°C, while in the PPT the water was at 18° ± 1°C, as we have found that this cold temperature engages an active escape behavior in the PPT. Despite the fact that the water was colder in the clockmaze, we found that MWM triggered a significantly larger drop in temperature, which was particularly noticeable in trials that lasted until the cut-off time of 60 s (Figure 9E; C57, *T*_(108)_ = 25.74, *P* = 6.8 × 10^−48^; BALB/c, *T*_(393)_ = 38.5, *P* = 1.77 × 10^−35^, *t*-test). For successful trials, lasting less than 60 s we found an inverse linear correlation between escape latency and temperature difference (Figure 9F; PPT, *ρ* = −0.76, *P* < 0.0001; MWM, *ρ* = −0.95, *P* < 0.0001, Spearman correlation).

## Discussion

This study has described a large-scale validation of the PPT as a robust paradigm to test spatial cognition in two strains of mice, C57 and BALB/c. Our work substantiates the seminal work by Deacon and Rawlins (2002), which demonstrated that mice with cytotoxic hippocampal lesions were deficient in learning the task. By applying a pharmacological approach, with bilateral intra-hippocampal muscimol injections (Figure 6), we further show that the dorsal CA1 region of the hippocampus is indeed essential for task acquisition. We used large cohorts of C57 mice (120 males, 40 females) and BALB/c mice (40 males, 90 females) to show that all subjects were quick learners in the PPT when finding a single exit across trials (Figure 2, one-exit test) or several exits that were switched daily (Figure 3, many-exits test).

Moreover, we have presented a simple and powerful method to segregate three potential escape strategies (spatial, chain and futile) that mice developed during the task. These escape strategies were established on the basis of escape latency and the number of errors, two easily quantifiable parameters conveying non-redundant pieces of information, and were further validated by CART modeling (Wickham, 2009; Kuhn et al., 2016; Ripley, 2016). Notably, escape latency mainly labeled failed trials (those that reach the cut-off time of 60 s) whereas a low number of errors (≤3) represented a sensitive marker for hippocampus-based spatial learning. A corollary of this observation is that the definition of a spatial strategy as a direct trajectory from start to goal (with zero errors) would be too rigid in the clockmaze, as it would label the vast majority of healthy animals as false negatives for hippocampus-based learning.

This study also described a counterbalanced comparison of the PPT and the MWM in C57 mice (20 males, 20 females) and BALB/c mice (20 males, 20 females). The yoked design showed that all subjects were able to acquire the PPT regardless of the testing order (Figure 8). Moreover, C57 males acquired the MWM efficiently either before or after the PPT. Intriguingly, C57 females showed a somewhat slower acquisition of the MWM when this was tested after the PPT, indicating a possible interference effect between tests (Figure 8E). BALB/c mice of both sexes were severely impaired in the MWM, during acquisition and the probe test, and this was independent of testing the MWM before or after the PPT (Figures 8B,D). Our study also examined how anxiety, exhaustion, and hypothermia influenced performance in the MWM and PPT. In the MWM, there was a clear anxiety scale such that BALB/c females > BALB/c males > C57 females > C57 males. BALB/c females were the most anxious group because they spent the highest amount of time in the periphery of the watermaze and had the highest CORT level (measured immediately after the last trial in the MWM). Of note, C57 males were the top performers in the MWM but still had high CORT levels after the last MWM trial, suggesting that even these good learners were stressed in the watermaze, a result that agrees with previous reports (Harrison et al., 2009). Conversely, the PPT did not seem to elicit high stress, as attested by the relatively low CORT levels in all groups (Figure 9C). With regard to the degree of exhaustion after the tests, the MWM clearly induced much higher fatigue when compared to the PPT in all animals (Figure 9D). Regarding hypothermia, there was a drop in body temperature of ~2°C in the PPT and ~5°C in the MWM for trials that lasted 60 s (Figure 9E), and an inverse correlation between body temperature and escape latency for the successful trials, indicating mild hypothermia in the PPT and marked hypothermia in the MWM. Thus, our results have confirmed the accepted notions that anxiety, exhaustion, and hypothermia constitute substantial interfering factors when running mice in the MWM (Francis et al., 1995; Whishaw and Tomie, 1996; Lipp and Wolfer, 1998; Iivonen et al., 2003).

Theoretical accounts of navigation have long posited that mammals, including humans, possess several mechanisms for navigating including one that is based on information about the position of the self relative to the environment (egocentric coding) and another that depends on the spatial relations between landmarks irrespective of the animal’s location or point of view (allocentric coding), thus forming a cognitive map of the environment (O’Keefe and Nadel, 1978; O’Keefe, 2007; Hartley et al., 2014). There is overwhelming evidence that the hippocampus is a crucial substrate for the allocentric cognitive map and, therefore, for accurate performance in spatial tasks (reviewed in Morris, 2007). Notably, many studies have shown that the hippocampus does not mediate all spatial strategies since animals with hippocampal lesions can rely on response-based egocentric strategies, which are likely encoded by cortical and striatal networks (Morris, 2007). In our study, the data from muscimol-treated animals (Figure 6) suggest that the PPT relies on hippocampus-based allocentric coding; however, further experiments are needed to fully demonstrate this point (Rogers et al., 2017).

A noteworthy feature of the PPT in the clockmaze is the seemingly low cost for the animal of “*not knowing but guessing,”* which reduces the operant aspect of the PPT and might be a potential factor as to why it is equally efficient in mice of different strains and sex. It could also account for the fact that mice can solve the many-exits test as efficiently as the one-trial test. In contrast to the MWM in which the animals have to swim constantly as they search for the hidden platform (or until the end of the trial if they are not successful), the PPT has a relatively low cost of not knowing because the mice simply paddle in shallow water, typically near the wall, until they find the true exit. Moreover, the cost of guessing is minimal in the PPT due to the proximity of the exits to each other, which may also explain why the animals tend to apply a mixture of spatial and chain strategies, as this combination can be considered highly efficient to solve the task.

In conclusion, we think the PPT coupled with escape strategy analysis represents a significant improvement as a bioassay to reliably and cost-efficiently assess hippocampus-mediated function in mice. Going forward, it may become a powerful screening tool associated with preclinical drug development to treat cognitive disorders.

## Data Availability

All datasets generated for this study are included in the manuscript and/or the Supplementary Files.

## Ethics Statement

All animal experimentation was performed in accordance with the National Institutes of Health (NIH) Guidelines, under protocols approved by the Institutional Animal Care and Use Committee (IACUC) of the Feinstein Institute for Medical Research. Our Animal Research Program is registered with the Department of Health and Human Services (DHHS), Office of Laboratory Animal Welfare (OLAW), United States Department of Agriculture (USDA #21R0107), Public Health Service (PHS #A3168-01) and New York State Department of Health (NYSDOH #A-060).

## Author Contributions

RS, TH, SR, YA-A, and PH designed the experiments. RS, TH, RK, TK, JS, SR, and PH performed experiments and analyzed the data. RS and SR performed the CART modeling. PH made the final figures. RS and PH wrote the manuscript. All authors approved the manuscript.

## Conflict of Interest Statement

The authors declare that the research was conducted in the absence of any commercial or financial relationships that could be construed as a potential conflict of interest.

## Footnotes

^1^https://www.r-project.org
