# Supplementary material for: Large-Scale Validation of the Paddling Pool Task in the Clockmaze for Studying Hippocampus-Based Spatial Cognition in Mice
Source: Front Behav Neurosci. 2019 Jun 7;13:121. doi: 10.3389/fnbeh.2019.00121 (PMC6568215; doi:10.3389/fnbeh.2019.00121)
Supplement: Supplementary file 1 [file Data_Sheet_1.PDF]

Supplementary Material

Supplementary Figure 1

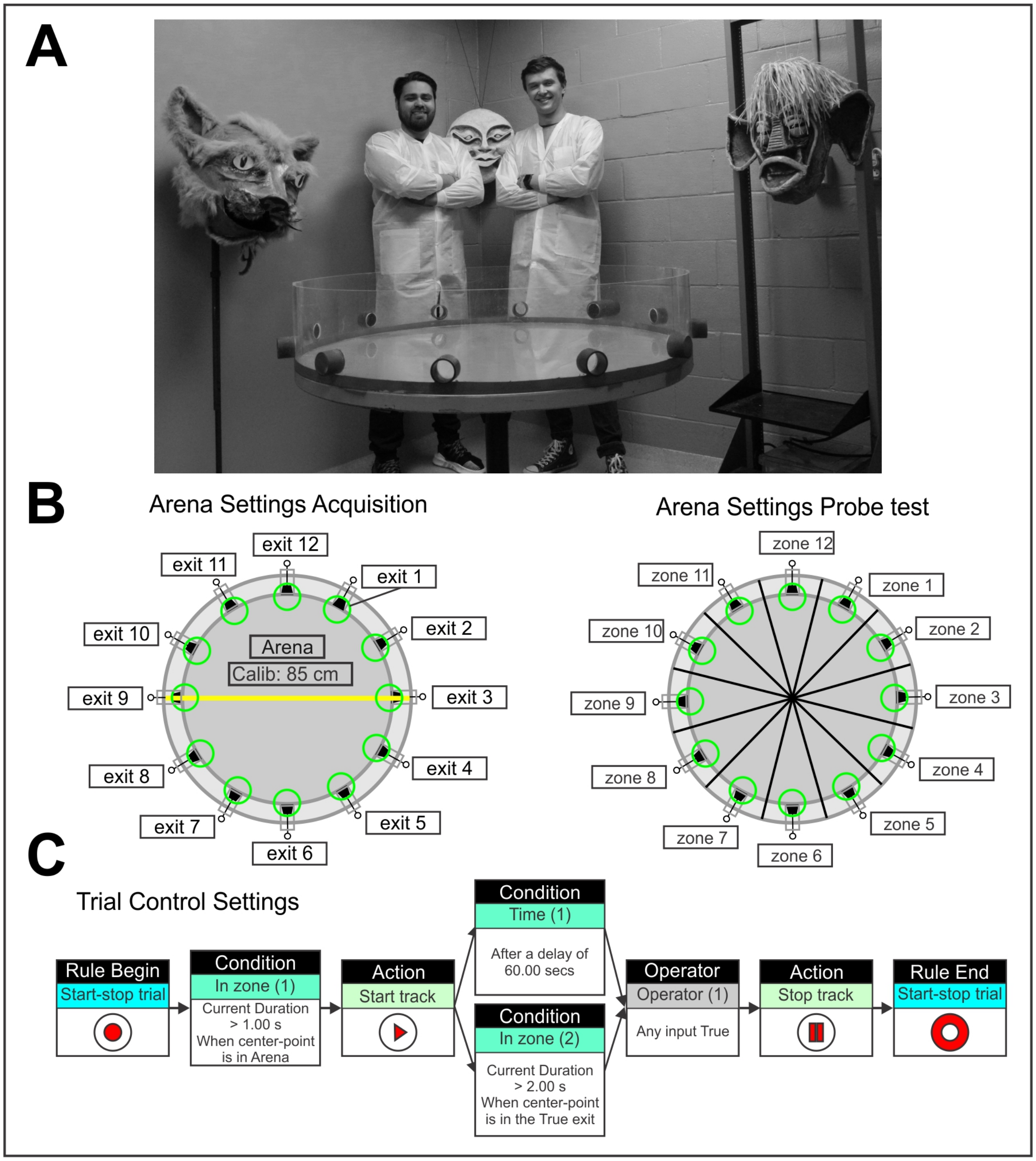

Implementation of the paddling pool task. (A) The testing area is 4m x 4m, with the apparatus (clockmaze, CM) sitting at the center of a round table, 1 m from the floor. A video camera is mounted in the ceiling and is connected to the video tracking system (Ethovision). Three large objects, consisting of 3-dimensional facemasks, are used as prominent distal cues. The testing area is illuminated at ~40 lux level by floor lights that are pointed to the distal cues from below. **(B)** <u>Guide</u>

<u>for the 'Arena Settings' in Ethovision</u>: the experimenter needs to take a background image of the CM, making sure that the entire maze fits within the scope of the camera. The arena is defined as a circle enclosing the outer perimeter of the CM, including the exit pipes (*left*). This will reduce potential background noise from the recording. Using the calibration rule tool (*left*), the diameter of the CM is properly assigned. Then, a Zone Group 1 is built, consisting of 12 circles with a radius of 2 cm positioned at the mouth of the exits. The circles are named Exit 1–12 so that they read like numbers on a clock. For the probe test (*right*), a Zone Group 2 is built by dividing the arena into 12 arcs, which are named Zone 1–12. **(C)** <u>Guide for 'Trial Control Settings' in Ethovision</u>: Add an "In Zone" condition prior to the stop condition, and link them together by dragging arrows between the boxes. Within the In Zone options, select only the true exit checkbox. Set the statistic current duration, and the time to $\geq$ 2s. Set the stop condition to a delay of 60s. When the true exit is changed, generate a new Trial Control Setting with the appropriate In Zone condition pertaining to the new exit zone. For probe trials, create a trial control setting without an In Zone condition and a stop condition of a 60s delay.
